# The Physiology, Pathology, and Therapeutic Interventions for ROCK Isoforms in Diabetic Kidney Disease

**DOI:** 10.3389/fphar.2020.585633

**Published:** 2020-09-25

**Authors:** Keiichiro Matoba, Yusuke Takeda, Yosuke Nagai, Kensuke Sekiguchi, Tamotsu Yokota, Kazunori Utsunomiya, Rimei Nishimura

**Affiliations:** ^1^ Division of Diabetes, Metabolism, and Endocrinology, Department of Internal Medicine, The Jikei University School of Medicine, Tokyo, Japan; ^2^ Center for Preventive Medicine, The Jikei University School of Medicine, Tokyo, Japan

**Keywords:** notch, hypoxia, inflammation, Rho (Rho GTPase), ROCK1/ROCK2, diabetic kidney disease (DKD)

## Abstract

Rho-associated coiled-coil-containing protein kinase (ROCK) is a serine/threonine kinase that was originally identified as RhoA interacting protein. A diverse array of cellular functions, including migration, proliferation, and phenotypic modulation, are orchestrated by ROCK through a mechanism involving cytoskeletal rearrangement. Mammalian cells express two ROCK isoforms: ROCK1 (Rho-kinase β/ROKβ) and ROCK2 (Rho-kinase α/ROKα). While both isoforms have structural similarities and are widely expressed across multiple tissues, investigations in gene knockout animals and cell-based studies have revealed distinct functions of ROCK1 and ROCK2. With respect to the kidney, inhibiting ROCK activity has proven effective for the preventing diabetic kidney disease (DKD) in both type 1 and type 2 diabetic rodent models. However, despite significant progress in the understanding of the renal ROCK biology over the past decade, the pathogenic roles of the ROCK isoforms is only beginning to be elucidated. Recent studies have demonstrated the involvement of renal ROCK1 in mitochondrial dynamics and cellular transdifferentiation, whereas ROCK2 activation leads to inflammation, fibrosis, and cell death in the diabetic kidney. This review provides a conceptual framework for dissecting the molecular underpinnings of ROCK-driven renal injury, focusing on the differences between ROCK1 and ROCK2.

## Introduction 

The World Health Organization estimates that, each year, around 1.2 million people worldwide die from end-stage renal disease (ESRD). Artificial kidneys and miniaturized dialysis save millions of lives, however dialysis requires cost up to US$91,000 per patient per year in the United States (End chronic kidney disease neglect, 2020), and fewer than half of those on dialysis survive for more than 5 years from the onset of ESRD. Diabetic kidney disease (DKD) in particular has had a devastating impact on the increasing frequency of ESRD.

One major breakthrough in the management of DKD came in the past two decades, when inhibitors of the renin-angiotensin system (RAS) were proven to attenuate the progressive impairment of the renal function. While cardiovascular outcome trials with sodium glucose co-transporter 2 (SGLT2) inhibitors demonstrated these agents’ renoprotective actions (Zinman et al., 2015; Kosiborod et al., 2017; Neal et al., 2017), the details are undoubtedly much more complex, with key concerns that current standards of care do not elicit complete remission. Given the limited drugs available to suppress DKD progression, there has been an ongoing effort to identify factors inducing renal injury and to develop effective therapeutic strategies.

Rho-associated protein kinase (ROCK) belongs to the family of serine/threonine kinases and is a major downstream effector of the small GTP-binding protein RhoA. ROCK signaling is involved in the regulation of a plethora of cellular functions. Due to its centrality in most cellular events, robust temporospatial and context-dependent regulation of ROCK is needed for cell homeostasis. In the kidney, over-activation of the ROCK pathway is clearly harmful; it promotes glomerular fibrosis and podocyte loss in the setting of a variety of diseases including but not limited to diabetes (Matoba et al., 2010; Meyer-Schwesinger et al., 2012; Matoba et al., 2013; Matoba et al., 2017). In addition, elevated ROCK activity results in the increase of oxidative stress, sodium retention, and vascular tone (Bussemaker et al., 2009; Calo et al., 2016; Calo et al., 2017). The beneficial effects of ROCK inhibition have been described in rodent models of DKD (Gojo et al., 2007; Kolavennu et al., 2008).

Two mammalian ROCK isoforms, ROCK1 (also known as Rho-kinase β/ROKβ) and ROCK2 (also referred to as Rho-kinase α/ROKα), have been identified (Nakagawa et al., 1996). The ROCK1 gene is located on chromosome 18 and consists of 1354 amino acids, while the ROCK2 gene is located on chromosome 2 and consists of 1388 amino acids. While these isoforms share 65% overall identity in amino acid sequence, ROCK1 and ROCK2 are differentially regulated, with distinct functions.

This review focuses on the pathophysiological functions of ROCK1 and ROCK2, and discusses the therapeutic effects of ROCK isoform inhibition in DKD.

## The Structure and Molecular Function of ROCK Isoforms

**Figure 1:**
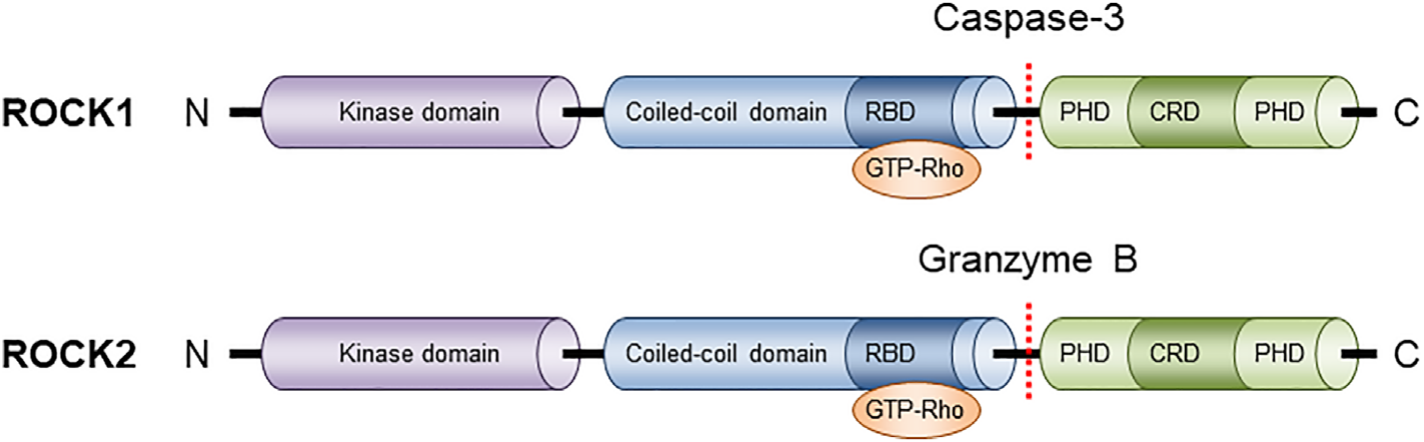
Structure of ROCK isoforms. ROCK1 and ROCK2 are known as ROKβ and ROKα respectively. Both isoforms consist of three major domains: a kinase domain in the N-terminal domain, a coiled-coil domain that contains a Rho-binding domain (RBD), and a putative pleckstrin homology domain (PHD) at its C-terminal end.

Among protein kinase neighbors, ROCKs are closely associated with myotonic dystrophy kinase-related Cdc42-binding kinase (MRCK) and citron kinase. These kinases have the same domain structure, which consists of an N-terminal kinase domain, a central coiled-coil region, and various functional motifs at their respective C-terminal (**Figure 1**). In ROCKs, these functional motifs contain Rho-binding domain (RBD) and pleckstrin homology domain (PHD) that is split into two by an internal cysteine-rich C1 domain (CRD). Under natural conditions, PHD blunts ROCK activity by sequestering kinase interface (Wen et al., 2008). Supporting of this is the fact that deletion of the C-terminal region including the PHD results in constitutive activation *in vitro* (Wen et al., 2008). However, when the RBD binds to GTP-bound active RhoA, RhoB, or RhoC, or PHD is removed, ROCK is constitutively activated. Despite the high sequence homology in their kinase domains, different machinery is involved in the activation process, with ROCK1 activated through the cleavage of the C-terminal PHD by caspase-3 and ROCK2 activation mediated by granzyme B-regulated cleavage. In addition, the inactivation process differs between these two isoforms: ROCK1 is negatively controlled by Rad GTP-binding protein, whereas ROCK2 is inhibited by Gem GTP-binding protein (Ward et al., 2002).

While ROCK1 is predominantly distributed in non-neural tissues including the gastrointestinal tract and lung, ROCK2 is found in the brain, kidney, and bladder (Nakagawa et al., 1996; Iizuka et al., 2012), indicating distinct actions of each isoform in these tissues. At the cellular level, ROCK1 has been detected in the cell membrane (Glyn et al., 2003), actin filaments, and lysosomes (Iizuka et al., 2012); however, the subcellular distribution of ROCK1 has not been fully clarified. ROCK2 activates p300 acetyltransferase to mediate gene transcription *in vitro*, which might explain why ROCK2 is predominantly localized to the nuclei (Tanaka et al., 2006). Consistently, ROCK2 is detected in euchromatin, where transcriptional events take place. ROCK1 and ROCK2 thus have different tissue and cellular distributions, which may affect their functions.

Findings obtained from global knockout of ROCK1 or ROCK2 have expanded our understanding regarding the function of each isoform. Mice harboring systemic ROCK1 deletion display impaired eye closure and an abnormal umbilical ring (Shimizu et al., 2005), whereas ROCK2 deficiency leads to intrauterine growth retardation (Thumkeo et al., 2003). While these data, coupled with other findings, suggest divergent physiological and pathological functions of ROCK isoforms, the specificity of those substrates has not been fully characterized (Hartmann et al., 2015).

## Mechanistic Insights Concerning ROCK Isoform Inhibition in DKD

**Figure 2:**
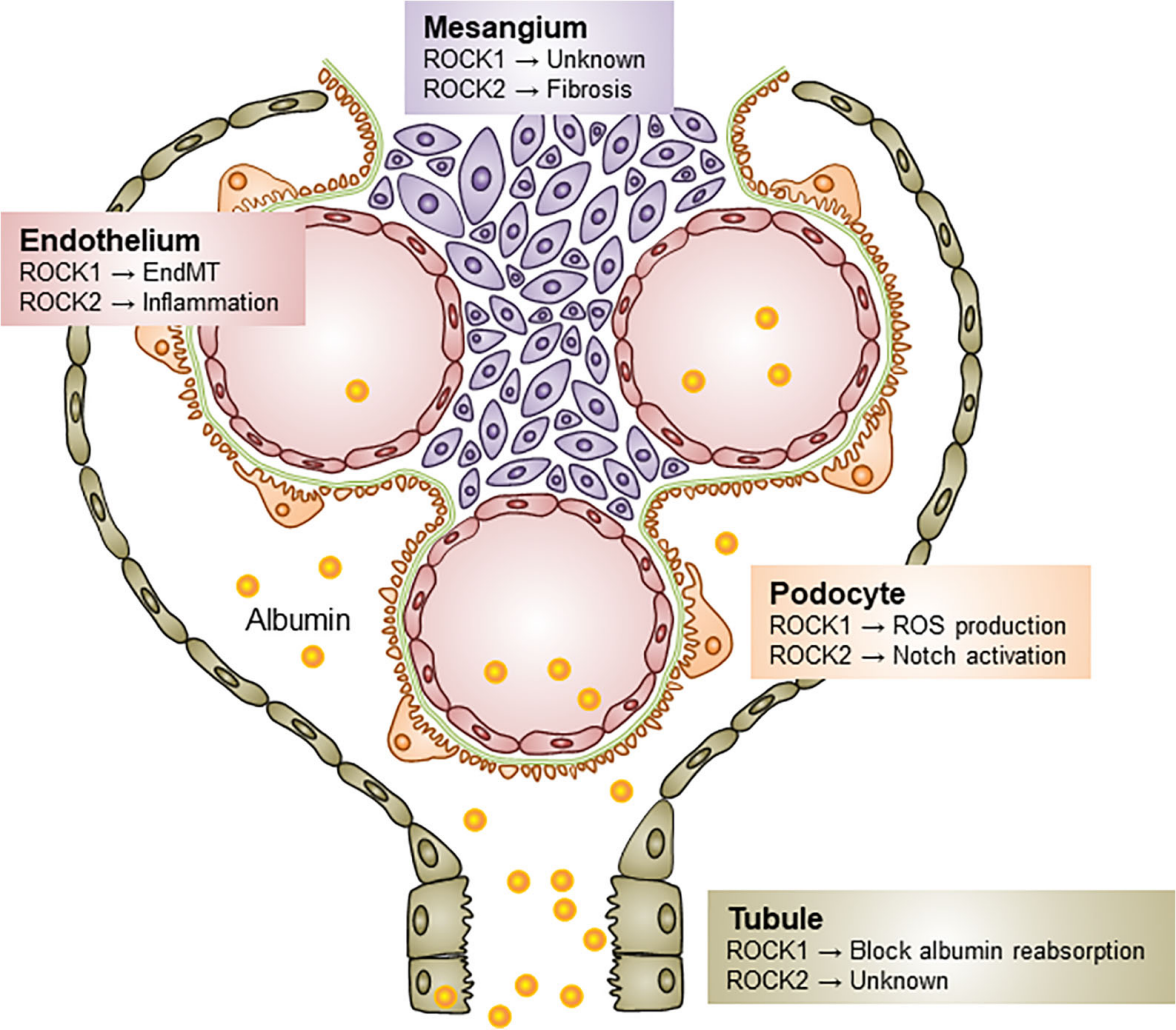
Distinct roles of ROCK isoforms in diabetic kidney disease. Both ROCK1 and ROCK2 contribute to the pathogenesis of DKD *via* different mechanisms. ROCK1 activation induces podocyte ROS production, EndMT, and blocks albumin endocytosis in tubular epithelial cells. Little is known about the role of ROCK1 on the mesangial biology, but ROCK2 elevation induces as the progression of mesangial expansion, Notch activation in podocytes, and endothelial inflammation. ROS, Reactive oxygen species; EndMT, Endothelial-to-mesenchymal transition.

Renal ROCK signaling is activated in rodent models of diabetes, regardless of the diabetes type (Gojo et al., 2007; Matoba et al., 2013). The ROCK-mediated molecular basis of DKD progression has been shaped by researchers using pharmacological inhibitors of ROCK (Y27632 and fasudil). Both of these agents ameliorate ROCK activity by competitively combining the ATP sites of the ROCK catalytic domain. While these studies have expanded ROCK research in the field of renal biology, these compounds inhibit both ROCK1 and ROCK2 with equal potency and have non-specific targets, such as protein kinase C, A, and mitogen-activated protein kinases at higher doses (Liao et al., 2007). Some of these disadvantages have been overcome by gene silencing approach, such as with small interfering RNA (siRNA) and systemic or conditional knockout. The distinct actions of each ROCK isoform in DKD are summarized in **Figure 2**.

## ROCK1-Mediated Albumin Transport, Mitochondrial Dynamics, Transdifferentiation in DKD

The upregulation of the ROCK1 isoform is detected in the glomerular endothelium and mesangium of db/db mice (Peng et al., 2016) as well as in the distal tubules of streptozotocin (STZ)-induced diabetic rats (Wu et al., 2013). In cell-based experiments, tubular ROCK1 is activated by the CXC chemokine ligand 16 (Liang et al., 2018), a cytokine produced by diabetic kidney (Ye et al., 2017), to drive production of pro-inflammatory cytokines including tumor necrosis factor α (TNF-α), interleukin 1β, and caspase-3 activation and apoptosis.

From a transcriptional standpoint, we previously showed that siRNA-mediated gene ablation of ROCK1 was sufficient to induce a reduction in hypoxia-inducible factor 1α (HIF-1α) under diabetic conditions (Matoba et al., 2013). In that study, the HIF-1α expression was also suppressed by ROCK2 inhibition, suggesting that both ROCK1 and ROCK2 are requisite for glomerular HIF-1α generation and downstream fibrotic reactions in mesangial cells. The specific action of mesangial ROCK1 has not yet been clarified.

A series of elegant and comprehensive investigations from the Danesh laboratory identified ROCK1-mediated molecular events in DKD using gain- and loss-of-function studies in mice (Wang W. et al., 2012). Intriguingly, ROCK1-deficient mice showed attenuation of albuminuria and histological abnormalities in these models. Conversely, podocyte-specific ROCK1 knockin confers a phenotype that has many of the features of DKD. Mechanistically, they described an unexpected direct action of ROCK1 for regulating mitochondrial fission through phosphorylation and the recruitment of dynamin-related protein-1 (Drp1). The results of that study implicate ROCK1 as a critical regulator of the mitochondrial dynamics in diabetes and suggest that ROCK1 may be a relevant therapeutic target for the generation of oxidative stress in podocytes.

The permselectivity of the glomerular filtration barrier limits the passage of albumin into the Bowman’s capsule, resulting in the loss of transport selectivity and culminating in albuminuria, as is common among individuals in DKD. Glomerular endothelium, a key component of the filtration barrier, is converted into the mesenchymal phenotype in cases of diabetes, a process termed endothelial-to-mesenchymal transition (EndMT). Peng et al. investigated the contribution of ROCK1 to EndMT using ROCK1-overexpressing glomerular endothelial cells (Peng et al., 2016). The authors performed quantitative polymerase chain reaction (qPCR) and Western blotting and observed the increased expression of mesenchymal markers (e.g. α-SMA and Snail), together with the loss of endothelial junctional molecules, particularly VE-cadherin. Collectively, they reported that the activation of ROCK1 triggers EndMT, resulting in the loss of cellular attachment to each other and vascular hyper-permeability. These data provide critical insights into the heretofore unclear functions of ROCK1 in the signaling pathway that mediates the damage to glomerular tight junctions and albuminuria in DKD.

Zhou et al. investigated the function of ROCK1 in STZ-induced DKD models (Zhou et al., 2011). To determine the pathological contribution of tubular ROCK1, the authors analyzed the phenotype of diabetic ROCK1-deficient mice. They found that genetic ablation of ROCK1 prevented the development of albuminuria, and this effect was associated with protection against the loss of megalin and cubulin, members of the low-density lipoprotein receptor family that mediate albumin endocytosis in proximal tubular epithelial cells (Zhai et al., 2000). That study provided novel insights into the role of ROCK1 in albumin reabsorption in tubules. Interestingly, benidipine, a calcium channel blocker, has been suggested to inhibit proteinuria by suppressing ROCK1 and the transdifferentiation of renal tubular epithelium without affecting the glucose metabolism or blood pressure (Wu et al., 2013).

The inhibition of both ROCK isoforms by Y27632 or fasudil is effective for preventing tubulointestinal fibrosis in unilateral ureteral obstruction (UUO) models (Nagatoya et al., 2002; Baba et al., 2015); however, the systemic deletion of ROCK1 did not protect against the obstructive kidney damage (Fu et al., 2006). There was no recovery of transforming growth factor β (TGF-β)/SMAD signaling or structural derangement in the kidney of ROCK1-deficient mice. As such, we may reasonably suggest that targeting ROCK1 alone may not be adequate for attenuating tubular fibrosis, at least in UUO models, and the pathological contribution of ROCK1 to tubules may differ between DKD and other renal disease.

Whether or not ROCK1 exerts other functions in DKD is not completely understood. Genome-wide screening approaches will be required to define ROCK1 targets and the precise mechanisms of action. Such analyses will also provide promising opportunities for the development of ROCK1 inhibitors and their translation into clinical medicine.

## ROCK2-Induced Fibrosis, Notch Activation, and Inflammation in DKD

Initial insights linking ROCK2 to diseases were gleaned from studies implicating ROCK2 as a regulator of, among others, immunity, inflammation, and fibrosis (Yang et al., 2018; Stam et al., 2019; Ricker et al., 2020). With regard to the kidney, we provided the first evidence indicating ROCK2 to be a core component of signaling circuitry that governs DKD progression. Nagai et al. demonstrated the upregulation of ROCK2 in the renal cortex of type 2 diabetic db/db mice (Nagai et al., 2019). In that study, ROCK2 inhibitors were evaluated for their efficacy against glomerular expansion and albuminuria *in vivo*. As a result, the preventive effects of these histological and functional abnormalities were confirmed. The authors also performed a loss of function analysis and revealed that gene deletion of ROCK2, but not ROCK1, decreased the fibrogenic response, concomitant with the suppression of phosphorylation of JNK and Erk, which in turn blocks the nuclear translocation of nuclear factor κB (NF-κB). Hence, ROCK2 inhibition appears to be a promising pharmacological intervention against DKD.

The podocyte slit diaphragm proteins nephrin and podocin are critical component forming the filtration barrier. In the context of diabetes, these components are damaged, mainly by the activation of Notch signaling pathways (Mathieson, 2011; Loeffler and Wolf, 2014). After the binding of Notch receptors to Notch ligands, such as Jagged-like and Delta-like, the C-terminal Notch intracellular domain (NICD) is cleaved from the cell membrane by γ-secretase and translocates into the nucleus, where the formation of recombination signal binding protein for immunoglobulin κJ region (Rbpj) and mastermind-like (MAML) proteins occurs in order to induce the expression of gene sets important for the development of the kidney (Malashicheva et al., 2020). The Notch pathway is reactivated in renal tissue obtained from diabetic mice to regulate the expression of Notch ligands (Niranjan et al., 2008). High-glucose conditions, TGF-β, or vascular endothelial growth factor (VEGF) are postulated to be the molecular basis for the upregulation of Notch signaling (Bonegio and Susztak, 2012). Of note, ROCK2-deficient podocytes are characterized by a significant reduction in TGF-β-induced Notch ligand expression (Matoba et al., 2017). In contrast, the induction of Notch ligand was not inhibited by ROCK1 gene deletion. These findings indicate the isoform-specific role of ROCK2 in podocytes and provide critical insights into potential strategies against albuminuria seen in DKD. Studies aimed at revealing the interdependency between ROCK2 and Notch modules through the generation of conditional knockout models are thus expected to be beneficial.

There is growing appreciation for the influence of vascular inflammation on regulating the progression of diabetic renal damage (Matoba et al., 2019). In addition to its effect in mesangial cells and podocytes, ROCK2 also plays important roles in endothelial cells. Takeda et al. conducted a series of studies to unravel the mechanisms by which ROCK2 activates vascular inflammation (Takeda et al., 2019). The qPCR array analysis of the mRNA expression profiles in ROCK2-null endothelium revealed differentially expressed genes related to vascular inflammation. Since chemokines and E-selectin production were downregulated in the endothelium, the authors examined monocyte migration and cell to cell adhesion, and found that these activities were abolished compared with those in endothelium with normal levels of ROCK2. These observations will need to be considered when establishing the contribution of ROCK2 to DKD, and when administering ROCK2 inhibitors to patients.

The impressive journey of ROCK2 inhibitors started with the development of KD-25 (formally SLx-2119), which is an orally available and selective inhibitor with a half maximal inhibitory concentration (IC_50_) and an inhibitory constant (Ki) of 60 nM and 41 nM, respectively (Boerma et al., 2008). Since this drug is used in clinical trials for patients with graft versus host disease (GVHD) and psoriasis (Yiu and Warren, 2016; MacDonald et al., 2017) (**Table 1**), ROCK2 inhibitors may could be used to treat DKD. The success of ROCK2 inhibitor clinical trials will hopefully inspire researchers to redouble their efforts to determine the molecular profiles responsible for ROCK2-regulated events in DKD.

**Table 1:** Clinical trials of ROCK inhibitors.

Disease	Interventions	Target	Phase	Status	Identifier	Primary outcome
Psoriasis	KD025 (SLx-2119) KD025 (SLx-2119) KD025 (SLx-2119)	ROCK2 ROCK2 ROCK2	2 2 2	Completed Completed Completed	NCT02106195 NCT02317627 NCT02852967	Safety and tolerability Safety and tolerability Number of subjects with a 75% decrease in PASI
GVHD	KD025 (SLx-2119)	ROCK2	2	Active, not recruiting	NCT03640481	Overall response rate
Systemic sclerosis	KD025 (SLx-2119)	ROCK2	2	Recruiting	NCT03919799	CRISS response
	Fasudil	ROCK1/2	3	Completed	NCT00498615	Skin temperature
Autoimmune disease/Fibrosis	KD025 (SLx-2119)	ROCK2	1	Completed	NCT03907540	Absolute bioavailability
	KD025 (SLx-2119)	ROCK2	1	Completed	NCT03530995	PK profile
Hepatic Impairment	KD025 (SLx-2119)	ROCK2	1	Recruiting	NCT04166942	PK profile
Chronic kidney disease	SAR407899A	ROCK1/2	1	Completed	NCT01485900	Number of patients reporting adverse events
Atherosclerosis	Fasudil	ROCK1/2	2	Completed	NCT03404843	Blood flow responses
	Fasudil	ROCK1/2	2	Completed	NCT00120718	Vascular reactivity
Diabetic macular edema	Fasudil	ROCK1/2	3	Completed	NCT01823081	Best corrected visual acuity
Retinopathy of prematurity	Fasudil	ROCK1/2	2/3	Recruiting	NCT04191954	Retinal vascularization
Glaucoma	Netarsudil (AR-11324)	ROCK1/2	1	Recruiting	NCT04234932	Peripapillary capillary perfusion density
Fuchs’ endothelial corneal dystrophy	Ripasudil (K-115)	ROCK1/2	4	Recruiting	NCT03249337	Corneal clearing
	Ripasudil (K-115)	ROCK1/2	2	Recruiting	NCT03813056	Time to corneal clearance
Amyotrophic lateral sclerosis	Fasudil	ROCK1/2	2	Recruiting	NCT03792490	Safety and tolerability
Erectile dysfunction	SAR407899	ROCK1/2	2	Completed	NCT00914277	Duration of penile rigidity during sexual stimulation

PASI, Psoriasis Area and Severity Index Score; CRISS, Combined Response Index in Diffuse Cutaneous Systemic Sclerosis (CRISS); GVHD, graft-versus-host-disease; PK, pharmacokinetics.

## Conclusions and Future Perspectives

Cardiovascular events are pertinent to morbidity and mortality in patients with DKD. Therefore, elucidation of molecular circuitry that governs atherogenic changes remains a major area of research. Recently, critical roles of ROCK isoforms in vascular disease have been evaluated by researchers. James Liao from Chicago and Hiroaki Shimokawa from Sendai are leaders in this field. Liao et al. identified macrophage ROCK1 as an essential element in the development of atherosclerosis through the modulation of foam cell formation and macrophage chemotaxis (Wang et al., 2008). ROCK2 also influences foam cell formation by inhibiting peroxisome proliferator-activated receptor-γ-mediated reverse cholesterol transport in inflammatory cells (Zhou et al., 2012). In vascular smooth muscle cells, ROCK2 controls migration and proliferation activities (Shimizu et al., 2013). In addition, Shimizu et al. focused on the pathologic role of ROCK2 in heart disease and showed that ROCK2 regulates hypertrophy of cardiomyocyte and cell death through interaction with serum response factor and ERK (Shimizu and Liao, 2016). These important findings coupled with the work of others have led to an increasing appreciation for ROCK2 as a critical molecule for not only renal disease but also cardiovascular disease.

As discussed above, published data have added to a burgeoning body of evidence that ROCKs are critical therapeutic targets against DKD and its related cardiovascular events. However, some caveats must be considered before this concept is accepted. First, the development of ROCK1-specific inhibitors and prospective intervention studies using ROCK1 or ROCK2 inhibitors are required in order to justify targeting ROCK isoforms to treat DKD. Second, whether an isoform-specific approach or pan ROCK inhibition would provide a better therapeutic outcome has yet to be clarified. The comparison of circulating and tissue levels of ROCK1 and ROCK2 between DKD patients and healthy subjects would facilitate our understanding the contribution of each isoform to the pathogenesis of DKD. These studies will also help identify useful targets of DKD therapy, which may vary by clinical stage, and allow for the earlier recognition of patients with diabetes who are at risk of DKD. Third, an open and thorough discussion of the risks while balancing potential clinical benefits of ROCK isoform inhibition is warranted. RhoA activation as well as RhoA inhibition results in podocyte damage (Wang L. et al., 2012), indicating that there is likely a narrow therapeutic window for ROCK isoform activity. This information will provide important insights to consider before commencing with ROCK isoform-selective inhibition in patients. In addition, given the impairment of insulin signaling in skeletal muscle observed in ROCK1 knockout mice (Lee et al., 2009), drugs with limited access to the kidney may be beneficial for patients with diabetes. However, it should be noted that the feasibility of ROCK inhibition has already been established with fasudil, a pan ROCK inhibitor, in patients with stroke (Shibuya et al., 2005). Moreover, clinical data of statins, which inhibit both ROCK1 and ROCK2 through the regulation of RhoA prenylation, demonstrate this medication to be well tolerated and safe during long-term treatment (Ford et al., 2016). Considering these findings alongside cogent evidence that ROCK is critical in versatile pathological aspects of diabetes, targeting ROCK1 and/or ROCK2 is expected to have therapeutic value for not only DKD but also other microvascular complications (i.e. retinopathy, neuropathy) (Yokota et al., 2007; Kanazawa et al., 2013). A deeper understanding of both the divergent and redundant roles of each isoform is therefore considered to be important for the development of effective therapeutic strategies, and for improving the prognosis of patients with diabetes.

## Author Contributions

KM wrote the manuscript. YT, YN, KS, TY, KU, and RN helped edit and revised the manuscript for important intellectual content. All authors contributed to the article and approved the submitted version.

## Funding

This work was supported by JSPS KAKENHI Grant Number 20K08645 and 18K15985 (to KM), the Yokoyama Foundation for Clinical Pharmacology (to KM), the MSD Life Science Foundation (to KM), the Takeda Science Foundation (to KM), the Suzuken Memorial Foundation (to KM), the Ichiro Kanehara Foundation (to KM) and the Japan Diabetes Foundation (to RN).

## Conflict of Interest

KM has received research support from Sanofi KK, Tanabe Pharma, and Takeda Pharmaceutical. RN has received speaker honoraria from Astellas Pharma, Nippon Boehringer Ingelheim, Eli Lilly Japan KK, Kissei Pharmaceutical, Medtronic Japan, MSD, Novartis Pharma KK, Novo Nordisk Pharma, Sanofi KK, and Takeda Pharmaceutical.

The remaining authors declare that the research was conducted in the absence of any commercial or financial relationships that could be construed as a potential conflict of interest.

## References

[B1] (2020). End chronic kidney disease neglect. Nature 579, 173. 10.1038/d41586-020-00691-4 32161393

[B2] Baba I. Egi Y. Utsumi H. Kakimoto T. Suzuki K. (2015). Inhibitory effects of fasudil on renal interstitial fibrosis induced by unilateral ureteral obstruction. Mol. Med. Rep. 12, 8010–8020. 10.3892/mmr.2015.4467 26498136 PMC4758322

[B3] Boerma M. Fu Q. Wang J. Loose D. S. Bartolozzi A. Ellis J. L. (2008). Comparative gene expression profiling in three primary human cell lines after treatment with a novel inhibitor of Rho kinase or atorvastatin. Blood Coagul. Fibrinol. 19, 709–718. 10.1097/MBC.0b013e32830b2891 PMC2713681 18832915

[B4] Bonegio R. Susztak K. (2012). Notch signaling in diabetic nephropathy. Exp. Cell Res. 318, 986–992. 10.1016/j.yexcr.2012.02.036 22414874 PMC3677813

[B5] Bussemaker E. Herbrig K. Pistrosch F. Palm C. Passauer J. (2009). Role of rho-kinase in the regulation of vascular tone in hypertensive renal transplant recipients. Atherosclerosis 207, 567–572. 10.1016/j.atherosclerosis.2009.05.025 19717154

[B6] Calo L. A. Vertolli U. Pagnin E. Ravarotto V. Davis P. A. Lupia M. (2016). Increased rho kinase activity in mononuclear cells of dialysis and stage 3-4 chronic kidney disease patients with left ventricular hypertrophy: Cardiovascular risk implications. Life Sci. 148, 80–85. 10.1016/j.lfs.2016.02.019 26872982

[B7] Calo L. A. Ravarotto V. Simioni F. Naso E. Marchini F. Bonfante L. (2017). Pathophysiology of Post Transplant Hypertension in Kidney Transplant: Focus on Calcineurin Inhibitors Induced Oxidative Stress and Renal Sodium Retention and Implications with RhoA/Rho Kinase Pathway. Kidney Blood Press Res. 42, 676–685. 10.1159/000483023 29131070

[B8] Ford I. Murray H. McCowan C. Packard C. J. (2016). Long-Term Safety and Efficacy of Lowering Low-Density Lipoprotein Cholesterol With Statin Therapy: 20-Year Follow-Up of West of Scotland Coronary Prevention Study. Circulation 133, 1073–1080. 10.1161/CIRCULATIONAHA.115.019014 26864092 PMC4894764

[B9] Fu P. Liu F. Su S. Wang W. Huang X. R. Entman M. L. (2006). Signaling mechanism of renal fibrosis in unilateral ureteral obstructive kidney disease in ROCK1 knockout mice. J. Am. Soc. Nephrol. 17, 3105–3114. 10.1681/ASN.2005121366 17005937

[B10] Glyn M. C. Lawrenson J. G. Ward B. J. A. (2003). Rho-associated kinase mitigates reperfusion-induced change in the shape of cardiac capillary endothelial cells in situ. Cardiovasc. Res. 57, 195–206. 10.1016/s0008-6363(02)00616-8 12504829

[B11] Gojo A. Utsunomiya K. Taniguchi K. Yokota T. Ishizawa S. Kanazawa Y. (2007). The Rho-kinase inhibitor, fasudil, attenuates diabetic nephropathy in streptozotocin-induced diabetic rats. Eur. J. Pharmacol. 568, 242–247. 10.1016/j.ejphar.2007.04.011 17511984

[B12] Hartmann S. Ridley A. J. Lutz S. (2015). The Function of Rho-Associated Kinases ROCK1 and ROCK2 in the Pathogenesis of Cardiovascular Disease. Front. Pharmacol. 6, 276. 10.3389/fphar.2015.00276 26635606 PMC4653301

[B13] Iizuka M. Kimura K. Wang S. Kato K. Amano M. Kaibuchi K. (2012). Distinct distribution and localization of Rho-kinase in mouse epithelial, muscle and neural tissues. Cell Struct. Funct. 37, 155–175. 10.1247/csf.12018 22986902

[B14] Kanazawa Y. Takahashi-Fujigasaki J. Ishizawa S. Takabayashi N. Ishibashi K. Matoba K. (2013). The Rho-kinase inhibitor fasudil restores normal motor nerve conduction velocity in diabetic rats by assuring the proper localization of adhesion-related molecules in myelinating Schwann cells. Exp. Neurol. 247, 438–446. 10.1016/j.expneurol.2013.01.012 23337773

[B15] Kolavennu V. Zeng L. Peng H. Wang Y. Danesh F. R. (2008). Targeting of RhoA/ROCK signaling ameliorates progression of diabetic nephropathy independent of glucose control. Diabetes 57, 714–723. 10.2337/db07-1241 18083785

[B16] Kosiborod M. Cavender M. A. Fu A. Z. Wilding J. P. Khunti K. Holl R. W. (2017). Lower Risk of Heart Failure and Death in Patients Initiated on Sodium-Glucose Cotransporter-2 Inhibitors Versus Other Glucose-Lowering Drugs: The CVD-REAL Study (Comparative Effectiveness of Cardiovascular Outcomes in New Users of Sodium-Glucose Cotransporter-2 Inhibitors). Circulation 136, 249–259. 10.1161/CIRCULATIONAHA.117.029190 28522450 PMC5515629

[B17] Lee D. H. Shi J. Jeoung N. H. Kim M. S. Zabolotny J. M. Lee S. W. (2009). Targeted disruption of ROCK1 causes insulin resistance *in vivo* . J. Biol. Chem. 284, 11776–11780. 10.1074/jbc.C900014200 19276091 PMC2673246

[B18] Liang H. Liao M. Zhao W. Zheng X. Xu F. Wang H. (2018). CXCL16/ROCK1 signaling pathway exacerbates acute kidney injury induced by ischemia-reperfusion. BioMed. Pharmacother. 98, 347–356. 10.1016/j.biopha.2017.12.063 29275176

[B19] Liao J. K. Seto M. Noma K. (2007). Rho kinase (ROCK) inhibitors. J. Cardiovasc. Pharmacol. 50, 17–24. 10.1097/FJC.0b013e318070d1bd 17666911 PMC2692906

[B20] Loeffler I. Wolf G. (2014). Transforming growth factor-beta and the progression of renal disease. Nephrol. Dial. Transplant. 29 Suppl 1, i37–i45. 10.1093/ndt/gft267 24030832

[B21] MacDonald K. P. Blazar B. R. Hill G. R. (2017). Cytokine mediators of chronic graft-versus-host disease. J. Clin. Invest. 127, 2452–2463. 10.1172/JCI90593 28665299 PMC5490762

[B22] Malashicheva A. Kostina A. Kostareva A. Irtyuga O. Gordeev M. Uspensky V. (2020). Notch signaling in the pathogenesis of thoracic aortic aneurysms: A bridge between embryonic and adult states. Biochim. Biophys. Acta Mol. Basis Dis. 1866, 165631. 10.1016/j.bbadis.2019.165631 31816439

[B23] Mathieson P. W. (2011). The podocyte as a target for therapies–new and old. Nat. Rev. Nephrol. 8, 52–56. 10.1038/nrneph.2011.171 22045242

[B24] Matoba K. Kawanami D. Ishizawa S. Kanazawa Y. Yokota T. Utsunomiya K. (2010). Rho-kinase mediates TNF-alpha-induced MCP-1 expression *via* p38 MAPK signaling pathway in mesangial cells. Biochem. Biophys. Res. Commun. 402, 725–730. 10.1016/j.bbrc.2010.10.093 20977889

[B25] Matoba K. Kawanami D. Okada R. Tsukamoto M. Kinoshita J. Ito T. (2013). Rho-kinase inhibition prevents the progression of diabetic nephropathy by downregulating hypoxia-inducible factor 1alpha. Kidney Int. 84, 545–554. 10.1038/ki.2013.130 23615507

[B26] Matoba K. Kawanami D. Nagai Y. Takeda Y. Akamine T. Ishizawa S. (2017). Rho-Kinase Blockade Attenuates Podocyte Apoptosis by Inhibiting the Notch Signaling Pathway in Diabetic Nephropathy. Int. J. Mol. Sci. 18. 10.3390/ijms18081795 PMC5578183 28820432

[B27] Matoba K. Takeda Y. Nagai Y. Kawanami D. Utsunomiya K. Nishimura R. (2019). Unraveling the Role of Inflammation in the Pathogenesis of Diabetic Kidney Disease. Int. J. Mol. Sci. 20. 10.3390/ijms20143393 PMC6678414 31295940

[B28] Meyer-Schwesinger C. Dehde S. Sachs M. Mathey S. Arefi K. Gatzemeier S. (2012). Rho-kinase inhibition prevents proteinuria in immune-complex-mediated antipodocyte nephritis. Am. J. Physiol. Renal Physiol. 303, F1015–F1025. 10.1152/ajprenal.00380.2011 22811486

[B29] Nagai Y. Matoba K. Kawanami D. Takeda Y. Akamine T. Ishizawa S. (2019). ROCK2 regulates TGF-beta-induced expression of CTGF and profibrotic genes *via* NF-kappaB and cytoskeleton dynamics in mesangial cells. Am. J. Physiol. Renal Physiol. 317, F839–F851. 10.1152/ajprenal.00596.2018 31364374

[B30] Nagatoya K. Moriyama T. Kawada N. Takeji M. Oseto S. Murozono T. (2002). Y-27632 prevents tubulointerstitial fibrosis in mouse kidneys with unilateral ureteral obstruction. Kidney Int. 61, 1684–1695. 10.1046/j.1523-1755.2002.00328.x 11967018

[B31] Nakagawa O. Fujisawa K. Ishizaki T. Saito Y. Nakao K. Narumiya S. (1996). ROCK-I and ROCK-II, two isoforms of Rho-associated coiled-coil forming protein serine/threonine kinase in mice. FEBS Lett. 392, 189–193. 10.1016/0014-5793(96)00811-3 8772201

[B32] Neal B. Perkovic V. Mahaffey K. W. de Zeeuw D. Fulcher G. Erondu N. (2017). Canagliflozin and Cardiovascular and Renal Events in Type 2 Diabetes. N Engl. J. Med. 377, 644–657. 10.1056/NEJMoa1611925 28605608

[B33] Niranjan T. Bielesz B . Gruenwald A. Ponda M. P. Kopp J. B. Thomas D. B. (2008). The Notch pathway in podocytes plays a role in the development of glomerular disease. Nat. Med. 14, 290–298. 10.1038/nm1731 18311147

[B34] Peng H. Li Y. Wang C. Zhang J. Chen Y. Chen W. (2016). ROCK1 Induces Endothelial-to-Mesenchymal Transition in Glomeruli to Aggravate Albuminuria in Diabetic Nephropathy. Sci. Rep. 6, 20304. 10.1038/srep20304 26842599 PMC4740844

[B35] Ricker E. Chinenov Y. Pannellini T. Flores-Castro D. Ye C. Gupta S. (2020). Serine-threonine kinase ROCK2 regulates germinal center B cell positioning and cholesterol biosynthesis. J. Clin. Invest. 3654–3670 10.1172/JCI132414 32229726 PMC7324193

[B36] Shibuya M. Hirai S. Seto M. Satoh S. Ohtomo E. (2005). Effects of fasudil in acute ischemic stroke: results of a prospective placebo-controlled double-blind trial. J. Neurol. Sci. 238, 31–39. 10.1016/j.jns.2005.06.003 16005902

[B37] Shimizu T. Liao J. K. (2016). Rho Kinases and Cardiac Remodeling. Circ. J. 80, 1491–1498. 10.1253/circj.CJ-16-0433 27251065 PMC5563468

[B38] Shimizu Y. Thumkeo D. Keel J. Ishizaki T. Oshima H. Oshima M. (2005). ROCK-I regulates closure of the eyelids and ventral body wall by inducing assembly of actomyosin bundles. J. Cell Biol. 168, 941–953. 10.1083/jcb.200411179 15753128 PMC2171774

[B39] Shimizu T. Fukumoto Y. Tanaka S. Satoh K. Ikeda S. Shimokawa H. (2013). Crucial role of ROCK2 in vascular smooth muscle cells for hypoxia-induced pulmonary hypertension in mice. Arterioscler. Thromb. Vasc. Biol. 33, 2780–2791. 10.1161/ATVBAHA.113.301357 24135024

[B40] Stam K. Cai Z. van der Velde N. van Duin R. Lam E. van der Velden J. (2019). Cardiac remodelling in a swine model of chronic thromboembolic pulmonary hypertension: comparison of right vs. left ventricle. J. Physiol. 597, 4465–4480. 10.1113/JP277896 31194256 PMC6852085

[B41] Takeda Y. Matoba K. Kawanami D. Nagai Y. Akamine T. Ishizawa S. (2019). ROCK2 Regulates Monocyte Migration and Cell to Cell Adhesion in Vascular Endothelial Cells. Int. J. Mol. Sci. 20. 10.3390/ijms20061331 PMC6471293 30884801

[B42] Tanaka T. Nishimura D. Wu R. C. Amano M. Iso T. Kedes L. (2006). Nuclear Rho kinase, ROCK2, targets p300 acetyltransferase. J. Biol. Chem. 281, 15320–15329. 10.1074/jbc.M510954200 16574662

[B43] Thumkeo D. Keel J. Ishizaki T. Hirose M. Nonomura K. Oshima H. (2003). Targeted disruption of the mouse rho-associated kinase 2 gene results in intrauterine growth retardation and fetal death. Mol. Cell Biol. 23, 5043–5055. 10.1128/MCB.23.14.5043-5055.2003 12832488 PMC162229

[B44] Wang H. W. Liu P. Y. Oyama N. Rikitake Y. Kitamoto S. Gitlin J. (2008). Deficiency of ROCK1 in bone marrow-derived cells protects against atherosclerosis in LDLR-/- mice. FASEB J. 22, 3561–3570. 10.1096/fj.08-108829 18556458 PMC2537434

[B45] Wang W. Wang Y. Long J. Wang J. Haudek S. B. Overbeek P. (2012). Mitochondrial fission triggered by hyperglycemia is mediated by ROCK1 activation in podocytes and endothelial cells. Cell Metab. 15, 186–200. 10.1016/j.cmet.2012.01.009 22326220 PMC3278719

[B46] Wang L. Ellis M. J. Gomez J. A. Eisner W. Fennell W. Howell D. N. (2012). Mechanisms of the proteinuria induced by Rho GTPases. Kidney Int. 81, 1075–1085. 10.1038/ki.2011.472 22278020 PMC3352980

[B47] Ward Y. Yap S. F. Ravichandran V. Matsumura F. Ito M. Spinelli B. (2002). The GTP binding proteins Gem and Rad are negative regulators of the Rho-Rho kinase pathway. J. Cell Biol. 157, 291–302. 10.1083/jcb.200111026 11956230 PMC2199248

[B48] Wen W. Liu W. Yan J. Zhang M. (2008). Structure basis and unconventional lipid membrane binding properties of the PH-C1 tandem of rho kinases. J. Biol. Chem. 283, 26263–26273. 10.1074/jbc.M803417200 18640982 PMC3258851

[B49] Wu G. Xu M. Xu K. Hu Y. (2013). Benidipine protects kidney through inhibiting ROCK1 activity and reducing the epithelium-mesenchymal transdifferentiation in type 1 diabetic rats. J. Diabetes Res. 2013, 174526. 10.1155/2013/174526 24364038 PMC3864155

[B50] Yang W. Zhou G. Yu T. Chen L. Yu L. Guo Y. (2018). Critical role of ROCK2 activity in facilitating mucosal CD4(+) T cell activation in inflammatory bowel disease. J. Autoimmun. 89, 125–138. 10.1016/j.jaut.2017.12.009 29269245

[B51] Ye Y. Chen Q. Li J. Jin L. Zheng J. Li X. (2017). CXCL16 deficiency attenuates diabetic nephropathy through decreasing oxidative stress and inflammation. Biochem. Biophys. Res. Commun. 491, 848–854. 10.1016/j.bbrc.2017.05.013 28478039

[B52] Yiu Z. Z. Warren R. B. (2016). Novel Oral Therapies for Psoriasis and Psoriatic Arthritis. Am. J. Clin. Dermatol. 17, 191–200. 10.1007/s40257-016-0179-3 26923915

[B53] Yokota T. Utsunomiya K. Taniguchi K. Gojo A. Kurata H. Tajima N. (2007). Involvement of the Rho/Rho kinase signaling pathway in platelet-derived growth factor BB-induced vascular endothelial growth factor expression in diabetic rat retina. Jpn. J. Ophthalmol. 51, 424–430. 10.1007/s10384-007-0471-0 18158592

[B54] Zhai X. Y. Nielsen R. Birn H. Drumm K. Mildenberger S. Freudinger R. (2000). Cubilin- and megalin-mediated uptake of albumin in cultured proximal tubule cells of opossum kidney. Kidney Int. 58, 1523–1533. 10.1046/j.1523-1755.2000.00314.x 11012887

[B55] Zhou L. Liu F. Huang X. R. Liu F. Chen H. Chung A. C. (2011). Amelioration of albuminuria in ROCK1 knockout mice with streptozotocin-induced diabetic kidney disease. Am. J. Nephrol. 34, 468–475. 10.1159/000332040 21986457 PMC3691875

[B56] Zhou Q. Mei Y. Shoji T. Han X. Kaminski K. Oh G. T. (2012). Rho-associated coiled-coil-containing kinase 2 deficiency in bone marrow-derived cells leads to increased cholesterol efflux and decreased atherosclerosis. Circulation 126, 2236–2247. 10.1161/CIRCULATIONAHA.111.086041 23011471 PMC3807088

[B57] Zinman B. Wanner C. Lachin J. M. Fitchett D. Bluhmki E. Hantel S. (2015). Empagliflozin, Cardiovascular Outcomes, and Mortality in Type 2 Diabetes. N Engl. J. Med. 373, 2117–2128. 10.1056/NEJMoa1504720 26378978

